# Correlation between Insertion Torque and Implant Stability Quotient in Tapered Implants with Knife-Edge Thread Design

**DOI:** 10.1155/2018/7201093

**Published:** 2018-05-15

**Authors:** Domenico Baldi, Teresa Lombardi, Jacopo Colombo, Gabriele Cervino, Giuseppe Perinetti, Roberto Di Lenarda, Claudio Stacchi

**Affiliations:** ^1^Department of Surgical Sciences, University of Genoa, Italy; ^2^Private Practice, Cassano allo Ionio, Italy; ^3^Private Practice, La Spezia, Italy; ^4^Department of Biomedical and Dental Sciences, Morphological and Functional Images, University of Messina, Italy; ^5^Department of Medical, Surgical and Health Sciences, University of Trieste, Italy

## Abstract

**Aim:**

To evaluate the correlation between insertion torque (IT) and implant stability quotient (ISQ) in tapered implants with knife-edge threads.

**Methods:**

Seventy-five identical implants (Anyridge, Megagen) were inserted by using a surgical drilling unit with torque control and an integrated resonance frequency analysis module (Implantmed, W&H). IT (N/cm) and ISQ were recorded and implants were divided into three groups (*n* = 25) according to the IT: low (<30), medium (30 < IT < 50), and high torque (>50). ISQ difference among groups was assessed by Kruskal-Wallis test, followed by Bonferroni-corrected Mann–Whitney *U*-test for pairwise comparisons. The strength of the association between IT and ISQ was assessed by Spearman Rho correlation coefficient (α = 0.05).

**Results:**

At the pairwise comparisons, a significant difference of ISQ values was demonstrated only between low torque and high torque groups. The strength of the association between IT and ISQ value was significant for both the entire sample and the medium torque group, while it was not significant in low and high torque groups.

**Conclusions:**

For the investigated implant, ISQ and IT showed a positive correlation up to values around 50 N/cm: higher torques subject the bone-implant system to unnecessary biological and mechanical stress without additional benefits in terms of implant stability. This trial is registered with NCT03222219.

## 1. Introduction

Dental implants are currently accepted as a predictable treatment option for the rehabilitation of both partial or total edentulism. Moreover, immediate and early loading protocols have been introduced into clinical practice in the attempt to shorten treatment time and minimize patient discomfort, with positive results [1]. During the early phases of healing, dental implants should be protected from detrimental micromovements [2, 3] which, according to the literature, should not exceed values ranging between 50 and 150 *μ*m to avoid risks for the osseointegration process [4, 5]. When exceeding this threshold, there is a concrete possibility that the bone-implant interface could be colonized by fibroblasts from the overlying connective tissue, with consequent implant encapsulation in fibrous tissue and clinical failure [6]. In this scenario, the role of primary stability has become extremely important and, in recent years, many studies focused on this crucial topic [7–9]. Primary stability is a surgical outcome due to the mechanical engagement between implant and host bone, being influenced by surgical technique and by fixture and recipient bed characteristics. Numerous noninvasive methods have been proposed to evaluate implant stability, including Periotest [10] and Dental Fine Tester and Implatest conventional impulse testing [11], but the most widespread techniques are implant insertion torque measurement (IT) [12] and resonance frequency analysis (RFA) [13]. Insertion torque is the measure of the frictional resistance encountered by the implant while moving forward apically through a rotatory movement on its axis. RFA is performed by measuring the response of a magnetic device screwed on the implant when excited by a vibration consisting of small sinusoidal signals. The peak amplitude of the response is recorded and encoded into a parameter called implant stability quotient (ISQ), ranging from 0 (minimum stability) to 100 (maximum stability).

The correlation between IT and ISQ has been investigated in numerous studies but it is still unclear: according to some authors the two parameters are in a direct relationship [14, 15], and other studies demonstrated no statistically significant correlations between them [16, 17].

Furthermore, it must be considered that implants with different characteristics show different biomechanical behaviors: changes in macrogeometry (tapered versus parallel-walled, thread shape, length, and diameter) and microgeometry (surface texture) lead to different IT and ISQ values even when inserted in the same osteotomic preparation [17–20]. It would be necessary to understand the individual response of each implant shape in terms of primary stability when inserted at different torques: the knowledge of the ideal IT could allow the clinician to better adapt site preparation procedure to the specific implant optimizing primary stability without applying unnecessary stress to the bone-implant system.

The aim of this multicenter prospective study is to evaluate the correlation between IT and ISQ in tapered implants with knife-edge thread design, inserted in human subjects.

## 2. Materials and Methods

### 2.1. Study Design

This multicenter prospective study has been conducted in accordance with the Good Clinical Practice Guidelines (GCPs) and following the recommendations of the Declaration of Helsinki as revised in Fortaleza (2013) for investigations with human subjects. The study protocol had been approved by the relevant Ethical Committee (Comitato Etico Regione Calabria, Sezione Area Nord, n°73/2016) and recorded in a public register (NCT03222219).

Every patient signed an informed consent form to document the comprehension of the protocol and of the objectives of this study (procedures, follow-up, and any potential risk involved). The patient has been authorized to make questions concerning the treatment and the study protocol and has been thoroughly informed about alternative therapies.

A meeting among the clinical centers was held before starting the research in order to illustrate the protocol and standardize surgical procedures. An operator for each clinical center received written information to standardize data collection and ensure reliable outcome reporting by different assessors.

The present study tested the null hypothesis of no difference in ISQ values among implants placed with different insertion torque values versus the alternative hypothesis of a difference.

### 2.2. Study Population

All patients treated by the clinical centers needing an implant-supported rehabilitation were eligible for entering this study. Patients underwent a thorough clinical examination to evaluate periodontal and occlusal parameters, and bone volume in the areas of interest was assessed by means of CBCT scan.

#### 2.2.1. Inclusion Criteria

- Indications for dental implant treatment, based on accurate diagnosis and treatment plan.
- Height of the residual bone crest in the programmed implant site ≥11 mm and thickness ≥ 6 mm.
- Healed bone crest (almost three months after extraction or tooth loss).
- Patient age > 18 years.
- Patients able to examine and understand the study protocol.
- Informed consent form.

#### 2.2.2. Exclusion Criteria

- Acute myocardial infarction within the past 2 months.
- Uncontrolled coagulation disorders.
- Uncontrolled diabetes (HBA1c > 7.5%).
- Radiotherapy to the head/neck district within the past 24 months.
- Immunocompromised patients (HIV infection or chemotherapy within the past 5 years).
- Present or past treatment with intravenous bisphosphonates.
- Psychological or psychiatric problems.
- Alcohol or drugs abuse.
- Full mouth plaque score >30% and/or full mouth bleeding score >20%.

#### 2.2.3. Surgical Protocol

**Figure 1:**
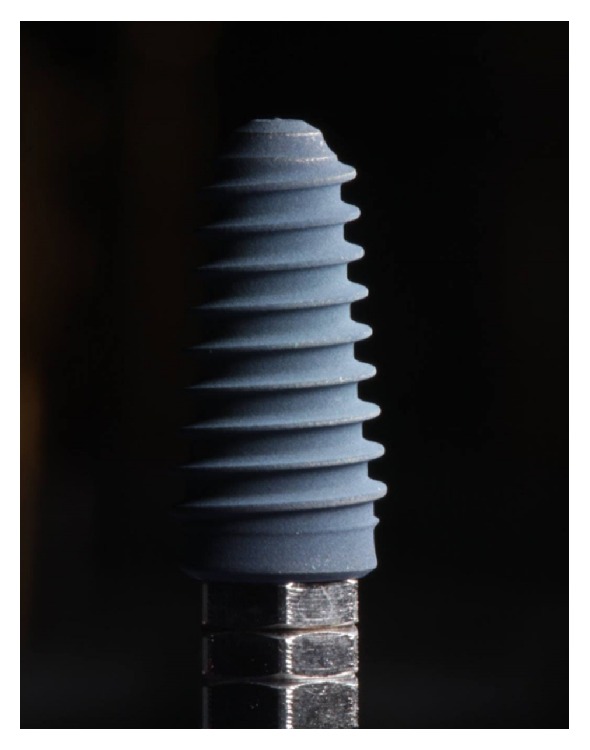
The investigational device was a 4 × 10 mm tapered implant with knife-edge threads (Anyridge, Megagen, South Korea).

Patients were asked to rinse with chlorhexidine mouthwash 0.2% for 30 seconds. Under local anesthesia (Artin, Omnia, Italy, articain 4% with adrenaline 1 : 100.000), a full thickness mucoperiosteal flap was elevated and initial osteotomy was performed by using an ultrasonic tip (S2, Piezomed, W&H, Bürmoos, Austria) for a better surgical control. Implant site preparation was then completed with the drills (2.0, 2.9, 3.3, and 3.8 mm diameter) of the selected implant system (Anyridge, Megagen, Gyeongsan, South Korea). A tapered implant with knife-edge threads was inserted (4 × 10 mm, Figure 1), following manufacturer recommendations (1 mm subcrestal placement).

**Figure 2:**
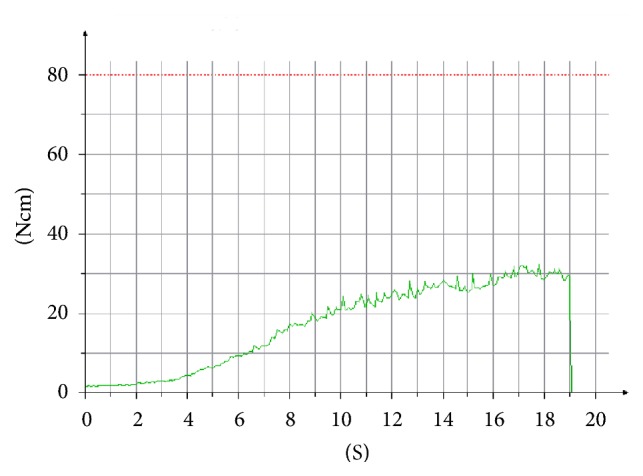
An example of insertion torque registration performed by the surgical drilling unit during entire placement. Ncm, newton/cm; S, seconds.

Implant insertion was performed by using a surgical motor with torque control and an integrated RFA module (Implantmed, W&H, Bürmoos, Austria). The unit recorded torque values (N/cm) during entire implant insertion on a removable USB memory stick (Figure 2). ISQ measurements were performed by a blinded examiner immediately after implant insertion, by using a specific disposable transducer (Smartpeg, Type 27). ISQ values were recorded in duplicate from mesiodistal, distomesial, buccolingual, and linguobuccal directions. Instrument calibration was verified before and after each patient visit, using an implant fixed in an epoxy resin block.

Implants were submerged under the soft tissues by suturing the surgical flap with Sentineri technique [21] and single stitches using a synthetic monofilament (PTFE, Omnia, Fidenza, Italy).

Patients were prescribed with antibiotics for 6 days (amoxicillin 1 g two times per day, or clarithromycin 250 mg two times per day for allergic patients) and nonsteroidal anti-inflammatory drugs (ibuprofen 600 mg), when needed.

#### 2.2.4. Postsurgical Follow-Up

Sutures were removed after ten days. After three months of submerged healing, implants were connected to healing abutments in order to start prosthetic procedures. Screwed ceramic crowns were delivered within 5 months after implant placement.

Finally, patients were inserted in a follow-up protocol with periodic professional dental hygiene recalls. Clinical and radiologic checks were performed 6 months and one year after implant loading to evaluate eventual complications.

### 2.3. Outcomes

This study evaluated the following outcome measures:

Primary outcomes:

- Insertion torque (IT): higher torque value (N/cm) recorded during implant placement.
- Implant stability quotient (ISQ): numerical value (0–100) recorded immediately after implant insertion and expressing resonance frequency analysis (RFA).

Secondary outcomes:

- Implant failure: implant mobility and/or any situation suggesting implant removal.
- Biological and mechanical complications: any complication defined as an unexpected deviation from the normal treatment outcome, both biological (e.g., mucositis, peri-implantitis) and mechanical (e.g., implant fracture, prosthesis fracture, and fixation screw loosening).

#### 2.3.1. Sample Size and Statistical Power

Treated patients were allocated into three groups, according to the peak torque value recorded during implant insertion: low torque (<30 N/cm), medium torque (30 < IT < 50 N/cm), and high torque (>50 N/cm). In patients treated with more than one implant, only the first inserted fixture was included in the subsequent analyses.

A sample of twenty patients for each group was necessary to achieve an effect size of 5 (±5) points on ISQ values (primary outcome), as a large effect indicator among the groups (*α* = 0.05 and power = 80%) (DSS Research, Fort Worth, USA). The effect size is defined as the difference in the given outcome between groups divided by the within-subjects standard deviations. Each clinical center treated 38 patients with the insertion of one dental implant for a total of 76 implants in order to compensate eventual drop-outs occurring during the study.

#### 2.3.2. Statistical Analysis

Equality of the groups by age and sex were evaluated by a one-way analysis of variance and a chi-squared test, respectively. For all the following analyses, patient was considered as the statistical unit.

Stability of each implant was described with a single ISQ value (mean of 8 measurements). The primary stability datasets were treated as ordinal because they did not meet the required assumptions for using parametrical methods (according to Kolmogorov-Smirnov and Levene tests). The significance of the difference in ISQ among groups was assessed by Kruskal-Wallis test, followed by Bonferroni-corrected Mann–Whitney *U*-test for pairwise comparisons. Moreover, the strength of the association between IT and ISQ was assessed by Spearman Rho correlation coefficient: this analysis was performed for the whole sample and within each experimental group.

The level of significance was set at *α* = 0.05.

## 3. Results

Seventy-five patients were enrolled and treated between June and September 2016 with the insertion of seventy-five conical implant with knife-edge threads (TL 41, CS 34).

Patients were allocated into three groups based on implant insertion torque values: low torque (<30 N/cm), medium torque (30 < IT < 50 N/cm), and high torque (>50 N/cm). Mean age was 61.6 ± 6.8, 57.5 ± 11.3, and 54.4 ± 13.2 in low, medium, and high torque group, respectively. Complete demographic characteristics of the three groups are listed in Table 1: in particular, groups were balanced by age and sex. In low torque group, 17 implants were inserted in maxilla and 8 in mandible; in medium torque group 12 implants were placed in maxilla and 13 in mandible; in high torque group 9 implants were positioned in maxilla and 16 in mandible (total 38 implants in maxilla and 37 in mandible).

IT and ISQ mean values were 18.8 ± 6.0 N/cm and 71.8 ± 6.6 in low torque group, 41.2 ± 7.2 N/cm and 75.6 ± 9.2 in medium torque group, and 68.2 ± 12.1 N/cm and 78 ± 6.4 in high torque group. At the pairwise comparisons, statistically significant difference among ISQ values was demonstrated only between low torque and high torque groups. Complete results are summarized in Table 2.

The strength of the association between IT and ISQ values resulted statistically significant both for the entire sample (*p* = 0.0001) and the medium torque group (*p* = 0.015), while it was not significant in the low and high torque groups (*p* = 0.094 and *p* = 0.565, resp.). Complete results are listed in Table 3.

After three months, two implants out of seventy-five (2.7%) were not osseointegrated: both implants were placed in the mandible and belonged to the high torque group (IT 80 N/cm and ISQ 79; IT 77 N/cm and ISQ 77, resp.). Seventy-three implants were loaded with screwed ceramic single crowns or bridges and all of them were satisfactorily in function at one-year follow-up. Three single crowns presented screw loosening during the follow-up period (two implants in high torque, one in medium torque group). No other biological or mechanical complications were recorded.

## 4. Discussion

The presence of sufficient primary implant stability, together with other factors like minimally traumatic surgical technique [22–25] and macro- and microgeometry of the fixture [26–28], is considered a crucial factor to obtain and maintain implant osseointegration. However, while these general concepts are currently widely accepted and recently confirmed by a recent review by Javed and Romanos [29], it is more challenging to define and control the different variables influencing the achievement of an adequate primary stability.

Although the final objectives of the surgery are common to all the implant systems, there is no universal technique for the preparation of the implant site. Many factors may contribute to the surgical stability of the fixture: preparation undersizing [30, 31], implant macrogeometry [19, 32], and microgeometry [30, 33], together with the qualitative and quantitative characteristics of the host bone (especially cortical thickness) [30], are the most relevant. As suggested by McCullough and Klokkevold [34], implant macrogeometry plays a fundamental role: variations in implant length, diameter, number of threads, thread depth, pitch, and helix angle may strongly influence primary stability [32]. This concept is currently widely debated and, as demonstrated by Lee et al. [35], implants with deeper thread depth provide higher primary stability, especially in low quality bone.

Santamaría-Arrieta and coworkers [16] showed that the other crucial variable is the surgical technique: in particular it is clear how, in general, the underpreparation of the implant site determines higher values of insertion torque, although it does not significantly affect primary stability [36]. It has also to be considered that excessive compression of the host bone, caused by high insertion torques, could result in a prolonged inflammatory phase: even if inflammation is always the necessary basis for tissue repair, a massive and long-lasting presence of proinflammatory cytokines could result in a delayed healing and marginal bone resorption [37–39]. Moreover, high insertion torques could cause permanent deformations of the implant platform (especially external hex connections), possibly jeopardizing long-term maintenance and stability of the entire prosthetic rehabilitation [40]. Furthermore, recent publications questioned the real need of reaching high IT values to achieve osseointegration: Verardi and coworkers [41] reported 100% medium-term survival rate of tissue level implants without primary stability at the time of insertion and Toljanic et al. [42] and Norton [43, 44] showed that implants with IT < 20 N/cm can yield favorable survival rates and optimal maintenance of marginal bone levels, even after immediate functional loading.

It seems evident from the aforementioned studies that implant site preparation needs to be individualized by evaluating bone quality (unfortunately still difficult to standardize) and the specific characteristics of the selected implant, in order to optimize the achievement of primary stability without unnecessary biological and mechanical stress to the bone-implant system. In the present study, in which implant site preparation followed a standardized protocol, the characteristics of the recipient site played a fundamental role: as expected, higher IT values were recorded in sites with dense cortical bone (especially mandible).

Several methods have been proposed to assess implant stability in an objective way. Insertion torque and RFA are the most widely accepted parameters and their relationship has been extensively analyzed by numerous researches. A recent systematic review, analyzing more than 2000 studies, concluded that insertion torque and RFA are independent and incomparable methods to measure primary stability [45].

The present study evaluated a tapered implant with knife-edge thread design and analyzed the variations of its primary stability measured by RFA when inserted with different torque values. The investigated implant demonstrated a satisfactory primary stability even when inserted with low torque values: mean ISQ of 71.8 was obtained in the group with a mean IT of 18.8 N/cm, confirming the findings on the same implant type reported by Lee et al. [35].

Our data demonstrated a general linear relationship between insertion torque and implant stability: the strength of this correlation resulted statistically significant for the entire sample (*p* = 0.0001), in accordance with a recent study by Zita Gomes et al. [46] on tapered implants with knife-edge thread design placed in the posterior maxilla. However, at a deeper analysis, the linear relationship between insertion torque and implant stability is valid only in the medium torque group (30 < IT < 50 N/cm) (*p* = 0.015), while it was not significant in low and high torque groups (*p* = 0.094 and *p* = 0.565, resp.). Therefore, the data from the present study suggested that, for the specific type of implant here selected, it seems reasonable to increase insertion torque up to 50 N/cm, in order to improve primary stability. At higher torque values, no significant further increase in primary stability could be demonstrated: mean ISQ values in medium and high torque groups did not differ significantly (75.6 and 78.0, resp.; *p* > 0.05). Moreover, a torque limited to 50 N/cm could be a protective factor from the potential risk of biological and mechanical complications related to the application of high torsional strengths [38–40]. In the present study, both lost implants (*n* = 2) were placed in the mandible with high IT (80 and 77 N/cm): according to literature, excessive bone compression could result in a significant reduction in bone-to-implant contact at the early phases of healing [47, 48] and in an increased implant failure rate [49].

The main limitation of this study was that present results are not automatically applicable to implants with different macro- and microgeometry from the investigational device here tested: each different implant shape (and also different implant length or diameter [50]) should be separately evaluated to establish the more convenient drilling protocol, optimizing primary stability without unnecessary biological and mechanical stress.

A second limitation consists in the current lack of a reliable method to define bone quality in a precise and measurable way: a sound and predictable definition of bone density could be an essential step both for researchers and surgeons to better adapt implant site preparation to the different clinical situations.

## 5. Conclusions

The need to standardize implant surgical techniques, combining an accurate knowledge of implant characteristics with a careful analysis of the surgical site, is a crucial topic in contemporary implantology. In particular, implant macro- and microgeometry and the possibility of achieving a predictable primary stability are important factors for long-term success of the therapy. With the limitations of this study, it can be concluded that the specific implant here tested presented a positive linear correlation between primary stability and implant insertion torque up to 50 N/cm: higher torque values could cause unnecessary stress to the bone-implant system without additional benefits in terms of stability.

## Figures and Tables

**Table 1:** Demographic characteristics.

Sex	Age				Sample numerosity			
	Low IT	Medium IT	High IT	Diff.	Low IT	Medium IT	High IT	Diff.
Males	58.7 ± 5.2	56.5 ± 13.4	55.5 ± 13.2	0.074^NS^	7	8	13	0.171^NS^
Females	62.7 ± 7.1	58.0 ± 10.6	53.2 ± 14.4		18	17	12	

Age is presented as mean ± standard deviation. IT, insertion torque. Diff. significance of the difference between the groups. ^NS^Difference not significant.

**Table 2:** Insertion torque (IT; in N/cm) and implant stability quotient (ISQ) according to the different groups.

Group	IT	ISQ
Low Torque	18.8 ± 6.0	71.8 ± 6.6
Medium Torque	41.2 ± 7.2	75.6 ± 9.2
High Torque	68.2 ± 12.1	78.0 ± 6.4^a^
Diff.	--	0.003^S^

Data are presented as mean ± standard deviation. *N* = 25 in each group. Diff., significance of the difference among the groups. Results at the pairwise comparisons. ^a^Significantly different from the low torque group. ^S^Statistically significant correlation.

**Table 3:** Spearman Rho correlation coefficient between insertion torque and implant stability quotient according to the different groups.

Group	Rho coefficient	Sig.
Low Torque	0.342	0.094^NS^
Medium Torque	0.481	0.015^S^
High Torque	0.121	0.565^NS^
Overall	0.461	0.0001^S^

*N* = 25 in each group. Overall refers to the whole sample. ^NS^Not statistically significant correlation. ^S^Statistically significant correlation.

## Data Availability

The datasets generated and analyzed during the current study are available from the corresponding author on reasonable request.
